# Interaction of alcohol and other potentially cardiotoxic substances with the cardiovascular system

**DOI:** 10.1007/s00059-024-05281-y

**Published:** 2024-11-27

**Authors:** Bernhard Maisch

**Affiliations:** 1 Philipps University Marburg and Herz- und Gefäßzentrum Marburg (HGZ), Marburg, Germany; 2 Feldbergstr. 45, 35043 Marburg, Germany

When seeing a patient for the first time, cardiologists and internists routinely ask about the individual’s basic health information such as body weight, height, age, and cardiovascular risk profile. The classic questionnaire on risk factors includes hypertension, diabetes, smoking, alcohol consumption, mobility, and physical activity or sports.

After going through this issue of *HERZ/Cardiovascular Diseases*, it becomes clear that this is only a *short list* of risk factors. This should be expanded to a *long list* of risk factors and specifically address the amount of weekly alcohol consumption and its combination with drugs and potentially cardiotoxic substances, which are often not revealed by patients during the first contact.

In his contribution on “Alcohol consumption—none is better than a little,” Bernhard Maisch revisits our previous attitude toward and evaluation of alcohol consumption until 2023, when a publication by Zhao et al. in that year[1] recalculated the relationship between the quantity of alcohol consumption and years of life lost. The cherished idea for decades was that a moderate consumption of alcohol could be beneficial for health and even reduce all-cause mortality, thus explaining the “French paradox” [2–4]. But their publication [1] led to a paradigm change and flattened the J‑shaped curve [5] to a straight line [6]. Drinking no alcohol at all is the healthiest option. One glass of beer or wine per week is tolerable [6]. This new orientation has influenced not only the recommendations of *Canada’s Guidance on Alcohol and Health* [6] but also the recommendations and guidelines of major health organizations. *HERZ/Cardiovascular Diseases* reported on this turnaround in 2023 [7].

The deleterious side of alcohol consumption is reflected in the fact that around 3 million people worldwide die every year as a direct or indirect result of alcohol use or misuse. But even more disturbing is the observation that the combined misuse with other drugs further increases the risk for carcinogenesis, mental health illness, and heart diseases. From the case report in his article on the abuse of both alcohol and cocaine (“crack”), it can be derived that the misuse of both substances can lead to severe rhythm disturbances, AV-block, and ventricular tachycardia, which even required the implantation of an automatic implantable defibrillator (AICD; [8]). Cocaine can cause arrhythmias either through the production of myocardial ischemia or as a direct result of ion channel alterations. The combined effect on sodium and potassium channel blocks and can give rise to a wide variety of supraventricular and ventricular rhythms. On the other hand, despite the common teaching that cocaine causes dilated cardiomyopathy, the meta-analysis by Arenas et al. did not identify enough primary data to conclude that either a single dose or chronic cocaine use can affect left ventricular function, apart from acute myocardial infarction (MI; [9]). However, cocaine together with the misuse of alcohol led to this detrimental consequences. Thus, the combined consumption of two substances such as alcohol and cocaine leads to the conclusion that both substances together may be even more dangerous than the mere sum of the two.

Hashish (from Arabic حشيش, ḥašīš, “grass”) refers to the resin that is extracted from parts of the female cannabis plant (Indian hemp). It is an extract often pressed into plates or blocks. Common synonymous terms for this are also “hash” or “shit.” Individual pieces of the pressed hashish plates are often called “piece.” Marijuana (colloquially also known as “grass, weed, pot, ganja or Mary Jane”) refers to the dried, resinous flowers and the small leaves of the female hemp plant (cannabis). Marijuana contains the psychoactive ingredient tetrahydrocannabinol (THC). Marijuana and hashish are both cannabis products; however, hashish usually consists of the pressed resin of the processed plant parts.

Cannabis and marijuana are the topic of two focused contributions in this issue. Julia Sharkova, Tanja Nickolaus, and Jürgen R. Schaefer put their main emphasis on the “Effects of cannabis use on the heart.” In Germany cannabis has been recently permitted for recreational use but the substance has also been used much longer for medicinal purposes. The negative effects on health and dependence have been recently disregarded in media and public discussions. The authors therefore paid special attention to undesired cardiovascular effects, as the cardiovascular system also reacts strongly to psychoactive substances. Examples are the induction of oxidative stress and inflammatory responses by cannabis smoking. An imbalance between free radicals and antioxidants can lead to cellular damage [10, 11]. The unwanted side effects were summarized in a recent telephone survey of 434,104 individuals between the ages of 18 and 74 years in the United States. Jeffers et al. found that a past 30-day cannabis use was associated with MI and stroke, and with composite outcomes of coronary heart disease (CHD), MI, and stroke among adults in a linear dose–response fashion [12]. The data were controlled for the status of tobacco smoking, age, sex, race and ethnicity, body mass index, diabetes, alcohol use, educational attainment, and physical activity. Their analysis showed a relationship in which more days of use were associated with higher risks. Among the younger men and women (< 65 years old) who had never used tobacco cigarettes and e‑cigarettes, cannabis use was significantly associated with stroke and the composite outcome of CHD, MI, and stroke.

Presenting another viewpoint on marijuana from a psychiatric angle, Johannes Kramer, Gabi Kolle, and Oliver Pogarell give us their professional insight into the psychiatric risks of cannabis use. Whereas desired effects are relaxation, calmness, or mild euphoria, cannabis or hashish consumption can also result in dependence with dysphoria or irritability, and it can trigger anxiety and depressive moods [13] and even psychosis [14].

Both contributions on cannabinoids emphasize the need for interdisciplinary collaboration between cardiologists and psychiatrists to effectively manage cannabis-related health problems. It has also become clear that the combined uses of marijuana and nicotine smoking are still poorly investigated.

Similar considerations apply to opium (from Greek ὀπος “juice”). Its Greek and colloquial name is derived from the dried milky sap of unripe seed pods of the opium poppy (Papaveraceae). This compound has been related to health problems for centuries, which is worth mentioning in this context, although it is not covered by a contribution of its own in this issue of *HERZ*. In the course of the drying process of opium, autooxidation produces a brown to black mass, the raw opium. The main active components of opium are the alkaloids morphine, codeine, and thebaine. Its trade name is “heroin,” which is the most widely used morphine derivative. Synthetic derivatives such as fentanyl and pethidine are medicinal products that act on the opioid receptors. They belong to the groups of opiates and opioids and are classified as opioids.

In their contribution on methamphetamine toxicity, Kristin Annawald, Katrin Streckfuss-Bömeke, and Thomas Meyer discuss the addictive potential of the drug. Methamphetamine is better known by the colloquial terms “ice” or “crystal meth.” Misuse occurs by oral, intravenous, and intranasal use, as well as by inhalation when smoking. Its deleterious effects on the cardiovascular system include hypertension, accelerated atherosclerosis, vasospasm-induced acute coronary syndromes, sudden cardiac death, and dilated cardiomyopathy with congestive heart failure. A massive methamphetamine exposure causes vasoconstriction and vasospasm, which may ultimately lead to hypertension, tachycardia, endothelial dysfunction, and cardiotoxicity.

The authors provide insight into the JAK-STAT3 pathway involved in ischemia/reperfusion injury in the myocardium, which may be activated by the vasospasm-inducing action of the drug, and they describe the role of the potentially cardioprotective STAT3 pathway in the pathogenesis of methamphetamine-induced cardiotoxicity [15, 16].

Fenethylline works on similar pathways as stimulant or *Weckamine*. Initially, it was produced as a medicinal drug under the tradename Captagon® and was prescribed to treat attention deficit disorder and narcolepsy. In the body it is metabolized to amphetamine and theophylline. The two main markets were Europe and the Middle East. Following global controls in 1986, the drug ceased to be produced for medical purposes. It has a long story as an illegal doping substance in sports and in soccer, in particular.

The World Health Organization reported that tobacco consumption is responsible for killing approximately half of its users, accounting for over 8 million deaths worldwide [17].

Whereas smoking of tobacco in cigarettes, cigars, and pipes has long been known as one of the leading causes of chronic noncommunicable diseases and a significant risk factor for cardiovascular and respiratory diseases, the socially accepted use of e‑cigarettes and vaping adds a new chapter to smoking as a well-known cardiovascular risk factor. Thomas Münzel, Marin Kuntic, Paul Stamm, and Andreas Daiber investigated the increasing use of e‑cigarettes and water pipes (shisha), particularly among younger individuals. Their conclusion is that e‑cigarette vaping does not offer a completely risk-free alternative to traditional cigarettes. But switching from traditional cigarettes to e‑cigarettes is associated with an improvement in endothelial function [18, 19]. The detrimental effects on endothelial function associated with shisha smoking and e‑cigarette vaping remain, however.

Obviously, the overview of dangerous toxicants for human health and the cardiovascular system in this issue of *HERZ* cannot be exhaustive. Nevertheless, it attempts to shed some light on recent developments in a changing environment of a transforming patient population. Truly, our *short list* of classic risk factors has to be expanded to a *long list*. And the long list will not end but will need to be expanded continuously. By the way, when we drink alcohol and make a toast with “prosit” or “to your health,” we paradoxically do the opposite of what we wish.

Yours

Bernhard Maisch
